# *In Silico* Single-Molecule Manipulation of DNA with Rigid Body Dynamics

**DOI:** 10.1371/journal.pcbi.1003456

**Published:** 2014-02-20

**Authors:** Pascal Carrivain, Maria Barbi, Jean-Marc Victor

**Affiliations:** 1 Laboratoire de Physique Théorique de la Matière Condensée, CNRS UMR 7600, Université Pierre et Marie Curie, Paris, France; 2 Institut de Génétique Humaine (IGH), CNRS UPR 1142, Montpellier, France; University of Missouri, United States of America

## Abstract

We develop a new powerful method to reproduce *in silico* single-molecule manipulation experiments. We demonstrate that flexible polymers such as DNA can be simulated using rigid body dynamics thanks to an original implementation of Langevin dynamics in an open source library called Open Dynamics Engine. We moreover implement a global thermostat which accelerates the simulation sampling by two orders of magnitude. We reproduce force-extension as well as rotation-extension curves of reference experimental studies. Finally, we extend the model to simulations where the control parameter is no longer the torsional strain but instead the torque, and predict the expected behavior for this case which is particularly challenging theoretically and experimentally.

## Author Summary

Video game techniques are designed to simulate rigid body dynamics of macroscopic bodies, e.g. characters or vehicles, in a realistic manner. However they are not able to deal with temperature effects, hence they are not able to deal with molecules. In order to extend these powerful techniques to molecular modeling, we implement here Langevin Dynamics in an open source library called Open Dynamics Engine. Moreover we add a “global thermostat” to this Langevin Dynamics, which accelerates the simulation sampling by two orders of magnitude. With these radically new simulation techniques, we prove that we can accurately reproduce single-molecule manipulation experiments in silico, in particular force-extension as well as rotation-extension curves of reference experimental studies. The method developed here represents an unparalleled tool for the study of more complex single molecule manipulation experiments, notably when DNA interacts with proteins. Furthermore the simulation technique that we propose here has all the functionalities required to tackle the nuclear organization of chromosomes at every length scale, from DNA to whole nuclei.

This is a *PLOS Computational Biology* Methods article.

## Introduction

The mechanical and topological properties of DNA and protein-DNA assemblies are of primary importance in many biological processes, including transcription, replication, chromatin organization and remodeling. Since techniques have become available enabling the manipulation of single-molecules [1], [2], a large amount of experimental data have been accumulated on the mechanical response of DNA and protein-DNA assemblies under stretching forces and twisting torsions, in particular from optical and magnetic tweezers experiments [2]–[5]. In magnetic tweezers experiments, a DNA molecule is grafted at one end to a coverslip and at the other end to a magnetic bead. The bead is trapped in the magnetic field of a pair of magnets that may be translated, thus exerting a varying force on the bead. Moreover the pair of magnets may be rotated at a certain number of turns, thus constraining the linking number of the DNA molecule. After the stretching force and the number of turns are applied to the bead, the only physical variable that can be directly measured is the DNA extension, *i.e.* the distance between its two ends. Therefore the interpretation of the experimental results requires an important modeling effort, particularly in the more complex cases where DNA is associated with proteins, as for instance in chromatin assemblies [6], [7]. Although theoretical approaches may be successful in some cases [8]–[10], simulations are often crucial tests of the proposed model validity, when they are not the unique possible way of dealing with the system complexity.

In this spirit, we aim to develop an efficient tool to manipulate single-molecules *in silico* reproducing optical and magnetic tweezers experiments. This task is challenging since the DNA model should have precise specifications to reproduce the behavior of DNA accurately. We need to: *(i)* model a polymer, i.e. an articulated chain; *(ii)* reproduce the effective diameter of DNA (depending on electrostatic conditions) and, when proteins are present, have the possibility to model their shape and steric hindrance; *(iii)* deal with collisions, especially in order to reproduce DNA supercoiled structures (plectonemes) and steric effects in DNA-protein assemblies; *(iv)* reproduce DNA twisting and bending elasticities; *(v)* include statistical mechanics features to account for temperature and thermal motion. Beside these essential points, we also wish to simulate the system *dynamics*, which may be important in some cases, e.g. when hysteresis is observed under magnetic tweezers [7] or for *in vivo* chromosome dynamics experiments in the cell nucleus [11], [12].

This ambitious list of specifications is beyond the reach of traditional simulation approaches where particles interact through 2-body potentials (as in Molecular Dynamics or Monte Carlo simulations [13], [14] with a given force field). The need to deal with frozen degrees of freedom in coarse grained modeling may be addressed through holonomic constraints, as in the SHAKE algorithm [15], [16], where an iterative approach is adopted. However, collision detection and steric hindrance may only be accounted for in this scheme by introducing additional steps. More recently, non iterative algorithms have been developed [17], that subsequently led to the development of new powerful tools, called “physics engines”. These have been designed by the engineering and robotics communities to reproduce the dynamic behaviour of articulated systems of rigid bodies. Physics engines are acquiring an increasing importance, notably in the fields of computer graphics and video games, where they are now widely used to simulate rigid body motion under realistic conditions and in real-time. Open Dynamics Engine (ODE) is one of the most popular rigid-body dynamics open source library for robotics simulation applications [18]. As other physics engines, ODE simulates the kinematics of articulated systems by using permanent joints that impose holonomic constraints, instead of bond potentials. The same method is used to manage collisions: when overlapping between bodies is detected, a temporary joint is locally created that reproduces the action of the contact forces, without the need for explicit permanent 2-body interaction potentials (see section “Materials and Methods” for details on how ODE manages joints and collisions).

These extremely efficient simulators haven't, up to now, been used in statistical mechanics. Although well adapted to mechanical simulations, physics engines lack coupling to a thermal bath. The main novelty of our approach is the implementation of Langevin-Euler equation in the ODE software. Moreover we improve the simulation efficiency of this Langevin dynamics by extending the “global thermostat” algorithm designed by Bussi and Parinello in 2008 [19] to physics engines. This algorithm allows much faster yet unbiased sampling of the phase-space. As a first step toward simulating DNA-protein assemblies, we focus here on bare DNA and show how to perform *in silico* single molecule manipulation of DNA.

## Materials and Methods

### Introduction to physics engines

In rigid body dynamics simulations run with ODE, the state of a system consisting of 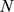 rigid bodies is described by the positions 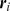 of their centres of mass, a quaternion representation of their orientations 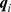, and their linear and angular velocities 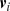 and 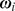 respectively. These velocities are collected in the column vector 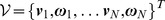. We use the superscript T to denote the transpose of a vector or a matrix everywhere in this article. The vector 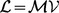 then collects all linear and angular momenta, where 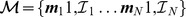 is a 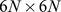 block diagonal matrix whose elements are the mass matrices 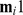 and inertia matrices 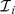 of the N bodies (with 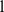 the 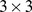 identity matrix). The Newtonian dynamics equation then reads 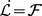 where the generalized force 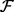 is a vector collecting forces and torques applied to the system. These forces and torques may be external, due for example to gravity or magnetic fields, or internal, as a consequence of the mechanical constraints between the rigid bodies that make up the system.

Most notably, in articulated systems, as is the case of polymers, rigid bodies are connected by mechanical joints. A joint is a relationship that is enforced between two bodies so that they can have only certain positions and orientations relative to each other, and ODE provides different types of joints according to the kind of articulation that has to be implemented, e.g. ball-and-socket, hinge, slider or universal.

Mathematically a joint imposes some holonomic constraint between both connected bodies. Such a constraint is an equation that reads 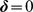 where 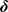 is the distance between both joint bearings, e.g. the center of the ball of one body and the the center of the socket of the other one. The constrained distance 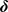 is purely geometrical, depending only on the relative position and orientation of both jointed bodies. The position and orientation of each of the 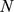 bodies the articulated system is composed of depend on time 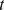. Therefore the constraints 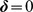 of the articulated system can be derived with respect to time to get the kinematic constraints in the form 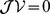 where we introduce the jacobian matrix of constraints 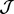 (see subsection “Exact solution for 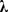 when there are no collisions” for a detailed example). This velocity-based description is used in ODE as in most game/physics engines.

So, mechanical joints exert reaction forces and torques on the joint bearings. These internal mechanical constraints can be collected into a generalized constraint force 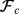 which, by virtue of the principle of virtual work 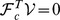, reads 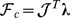 where 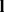 is a vector of Lagrange multipliers that precisely accounts for the reaction forces and torques coming from the joint bearings [16]. The Newtonian dynamics equation therefore reads 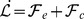 where 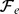 and 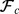 stand for the external and internal contributions to the generalized force respectively. As the constraint force reads 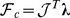, the Newtonian dynamics equation becomes an equation for 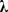 in the form: 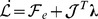.

Solving this equation for 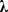 should moreover satisfy the holonomic constraints 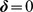 at every timestep 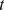. However the discretization used in the numerical calculation results in errors on 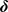 so that 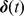 is generally not equal to 0. Then, in order to have 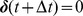 at the next timestep, the kinematic constraint 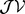 should be adapted accordingly. Indeed 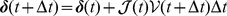 according to the Euler semi-implicit integration scheme which is used in velocity-based algorithms. Hence 
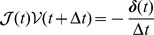
. But then this implies that the kinematic constraint is not equal to zero at time 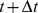, i.e. 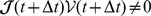, so that the joint bearings will continue to move apart afterwards. In order to keep both 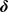 and 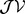 close to zero at every timestep, ODE introduces an error reduction parameter 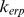 in the kinematic equation 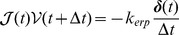 [18]. This parameter has to be adjusted to some optimal value between 0 (no correction at all) and 1 (complete correction of 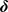 in one timestep). However setting 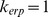 is not recommended since, as said above, this would imply that the joint bearings will continue to move apart afterwards with maximal velocities. ODE recommends values between 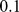 and 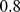.

**Figure 1:**
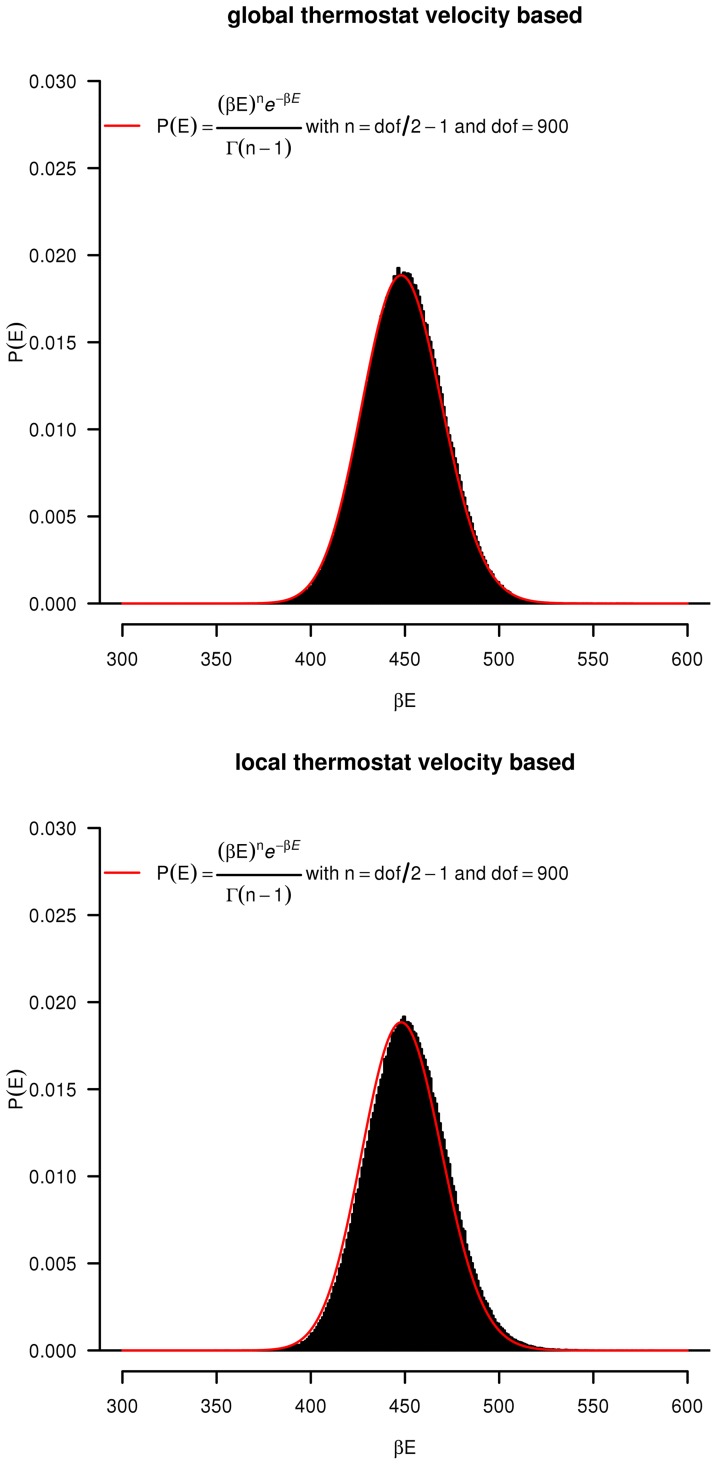
Boltzmann statistics test. The distribution of kinetic energy 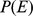 of a DNA molecule of length 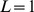 µm at thermal equilibrium is plotted as a function of the dimensionless kinetic energy 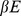 for both global (left panel) and local (right panel) thermostats. The DNA molecule is composed of 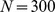 rigid cylinders of radius 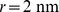 and 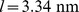. The other parameters of the simulation are given in Table 1. According to Eq. (33) the number of degrees of freedom is 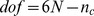 with 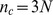 the number of non-redundant holonomic constraints (3 per ball-and socket joint, hence 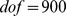). The factor 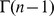 ensures the normalization of 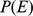 (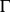 is the Euler Gamma function).

In addition to 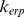, ODE introduces a second ingredient to soften the rigid constraints by allowing the violation of the constraint equation by an amount proportional to the restoring force 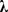. More explicitly, a “constraint force mixing” diagonal matrix 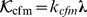 is defined, such that 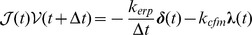 (implicit integration) [18]. This is equivalent to introducing a spring-damper system (spring constant of 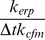 and damping constant of 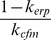) with implicit integrator between the joint bearings; this can be understood as analogous to a bead-spring model. Nevertheless there is a major difference between this effective spring and a regular spring: the term 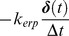 constrains the velocity whereas a regular spring constrains the acceleration. As a result, no energy is stored in this effective spring, at odds with a regular spring which stores an averaged energy 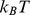 (see below subsection “Preliminary tests of validity and performance of the global thermostat” along with the histograms of energy in Figure 1).

In particular, ODE uses a powerful software called *libccd* [20] to detect collisions between two convex shapes. Whenever overlapping is detected between two rigid bodies, ODE attaches a temporary joint between them called a “contact joint”. Defining vector 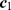 (resp. 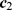) that connects the center of mass of body 1 (resp. 2) to the contact point and denoting 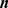 the common normal to both bodies at the contact point (directed from 2 to 1), the kinematic constraint imposed by the contact joint would read 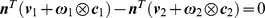 in the perfect case when the holonomic constraint imposed by the contact joint reads exactly 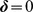. However, in practice 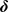 is not equal to 0 because of discretization errors, hence the kinematic constraint imposed by the contact joint actually reads:
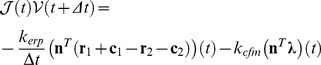
(1)with
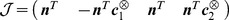
(2)

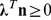
(3)


The right hand side of Eq.(1) deals with the already existing overlapping of the two bodies in contact at time 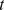 when collision is first detected, or with their residual overlapping while the contact joint exists.

By inserting the constraint force 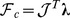 into the equation of motion 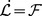 and taking the first-order discretisation of this equation, one can easily get the following expression to be solved for 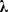:
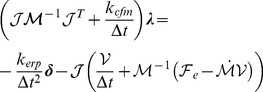
(4)


This equation is of the form 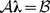. Importantly, the addition of the term 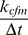 to each diagonal term **o**f the matrix 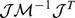 provides a symmetric positive definite matrix 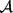, thus greatly increasing the solution accuracy of Eq.(4). From this equation, the vector 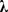 of Lagrange multipliers, hence the constraint force 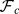, can be determined. Then the motion solver (semi-implicit Euler integrator) gives the new positions and orientations of the articulated bodies at time 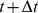. It is advantageous to choose the Exponential Map parametrization [21] for the quaternion integration.

### Solving constraints

In general Eq.(4) has to be solved numerically and ODE has two algorithms to do so, one based on the Successive-Over-Relaxation (SOR) method [22] and the other based on the Linear Complementary Problem (LCP) [23]. LCP time complexity is of order 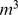 and space complexity (memory) of order 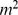 where 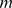 is the number of constraint rows [18]; whereas SOR time complexity is of order 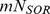 where 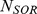 is the number of successive-over-relaxation and space complexity of order 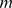 [18]. Both algorithms have equivalent performances when 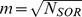. But in general LCP is more accurate, although much more time consuming, than SOR. We compared these two algorithms for a chain of length 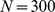 without noticing significant differences in the errors on 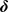 (error on the colocalization of joint bearings). In order to save computational time, we preferentially run the SOR method with a value of 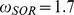 for the relaxation factor and 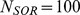. These values are different from the default values in ODE and work well for a linear chain of rigid bodies connected with ball-and-socket joints. But in some cases, the SOR method does not converge and we then switch to the LCP method, which always converges. However in the case when there are no collisions between the rigid bodies the articulated system is composed of, we were able to derive an exact solution for 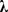 (see next subsection). Therefore we solve Eq.(4) according to the following scheme:

1. if there are no collisions at time 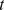, we use the exact solution for 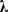,
2. if some collisions are detected, then we run the SOR method and check for the accuracy of the solution. More specifically the solution is accepted if 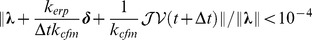,
3. if 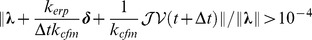 then the simulation step is restarted with the LCP method.

### Exact solution for 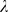 when there are no collisions

For the sake of clarity, let us first consider the example of four rigid cylinders of length 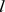 each connected with ball-and-socket joints at the extremities 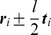 with the first one anchored to some fixed point, taken as the origin of the coordinates. The vector 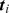 is the tangent to the cylinder 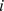. The jacobian matrix 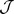 associated with this system is tridiagonal when there are no collisions, in which case it reads:
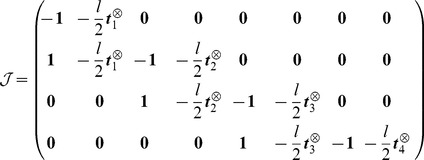
(5)


For each cylinder, the antisymmetric matrix 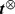 is associated with the cross product 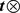 and has the property 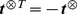:
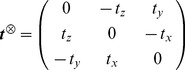
(6)


The transpose Jacobian matrix 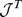 and mass matrix 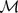 are given by:
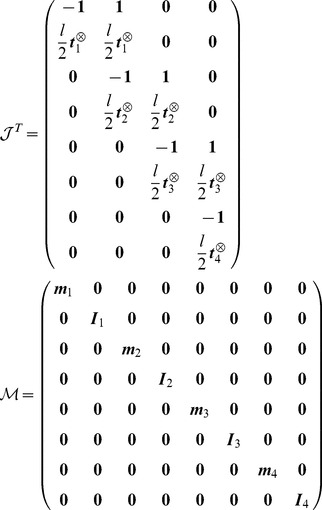
(7)


Then we get:
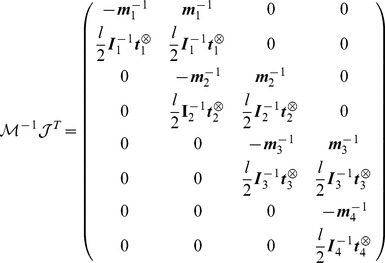
(8)and we deduce the final result for the symmetric matrix 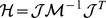:
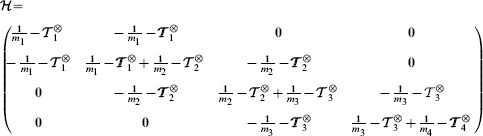
(9)where we define the matrix 
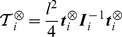
. We then write 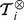 in the associated principal axis body frame as 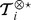:
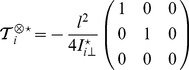
(10)


The vector 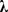 collects the Lagrange multipliers associated to each joint respectively 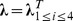. The equation 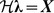 gives us a system of coupled equations on 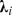. Note that ODE solves in one time all the constraints of this articulated system. This is not the case with the SHAKE algorithm where an unconstrained step is first performed, before correcting the positions and orientations iteratively to get the constraints satisfied eventually. The term 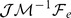 from Eq.(4) is given by:
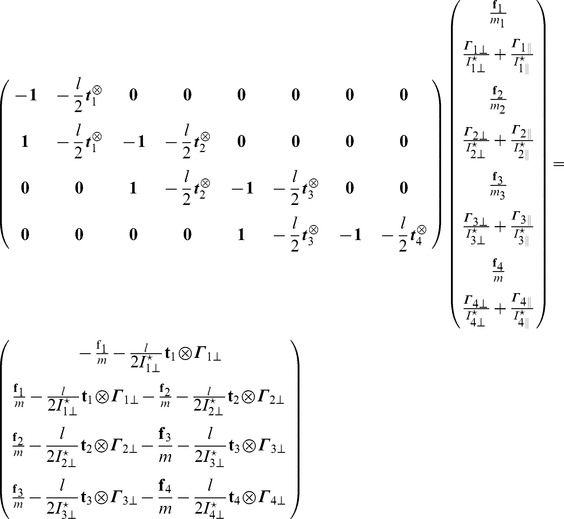
(11)where we write 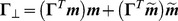 and 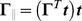. We can then write the constraint forces and torques 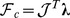


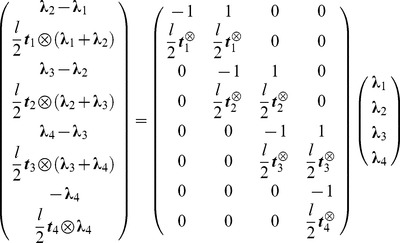
(12)


We can now generalise the previous example to the case of a linear chain of 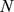 rigid cylinders connected with ball-and-socket joints with the first one anchored to the ground. We denote 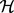 the matrix 
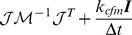
 with the following properties:
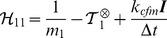
(13)

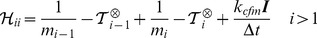
(14)

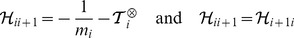
(15)

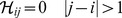
(16)


Using the following decomposition 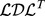 for the matrix 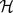 where 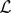 is a block lower matrix with block identity matrix on the diagonal and where 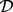 is the block diagonal matrix it is easy to show that 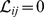 for 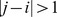. From these we get the following equations:
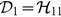
(17)

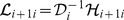
(18)

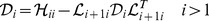
(19)


In order to solve the linear system of equations 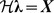 we define 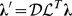 and solve the problem 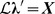 in an iterative way:
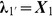
(20)

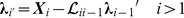
(21)and we get the final solution for 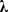 by solving the problem 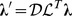:
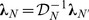
(22)

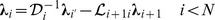
(23)


The method explained here is the exact solution of the problem 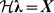 where no collisions are present in the system. With this exact resolution the simulation is faster than the SOR algorithm (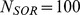 and 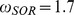) with a gain of 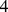.

### Simulating DNA molecules under magnetic tweezers

To model a DNA molecule, we build a linear chain of rigid cylinders of length 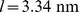 each, corresponding to 10 base pairs (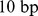), which amounts to the double helix pitch. We connect the cylinders to each other by ball-and-socket joints. The radius of the cylinders is set according to the salt buffer concentration of the experimental data we compare with. Indeed, since the DNA molecule is highly negatively charged, DNA-DNA electrostatic repulsion affects the double helix response in single molecule experiments [9], [24]–[27]. This effect can be easily and implicitly included in simulations and theoretical models by introducing an effective DNA radius 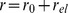 where 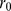 is the crystallographic radius of the DNA double helix and 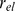 accounts for the DNA-DNA electrostatic repulsion [28]–[31]. It turns out that 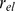 may be set equal to the Debye length 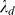 of the salt buffer solution. As 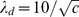 with c the salt concentration given in 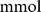 and 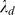 in 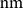, we set the effective radius to 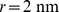 in 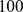
mmol monovalent salt buffer for comparison with the reference experimental data of Mosconi et al [32]. Alternatively we use an effective radius 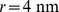 to fit the experimental data obtained in 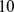
mmol monovalent salt buffer by Smith et al [1].

We performed all our *in silico* single molecule experiments with a DNA molecule of contour length 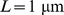. The corresponding number of DNA cylinders in the chain is therefore 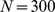. The DNA molecule is anchored, at one end, to a planar surface (mimicking the microscope coverslip), and at the other end, to a rotatable bead (mimicking the magnetic bead). We set the bead radius to 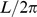 in order to prevent the DNA from looping around it. At both ends of the DNA chain, the rigid cylinders are tangent to their attachment surface.

The final problem that remains to be addressed is how to obtain a correct definition of the bending and twisting behavior of DNA. We have solved this problem by a special choice of the connecting joints and by introducing appropriate restoring torques reacting to the bending and twisting deformations. This has been done based on the bending and twisting energies that are defined according to the usual expressions 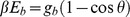 and 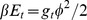 respectively. The rigidity constants 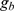 and 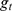 are related to the bending and twisting persistence lengths 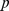 and 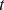 respectively, through the following equations:
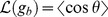
(24)

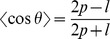
(25)

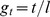
(26)where 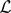 is the Langevin function (see supplementary Text S1). The bending angle 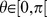 and twisting angle 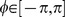 are related to the standard Euler transformation ZXZ and are given by
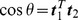
(27)

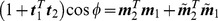
(28)

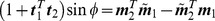
(29)where 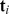 is the tangent vector of cylinder i, 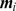 a vector normal to 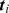 and 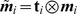. These three vectors are the principal axis of cylinder i.

We finally get the following expression for the global restoring torque between two connected DNA segments (see supplementary Text S1 for the complete derivation of this equation):
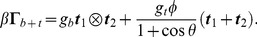
(30)


We recall that, for DNA, estimates of the bending persistence length give 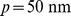 for 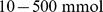 salt buffer (see Refs. [1], [33]–[35]); whereas estimates of the twisting persistence length give 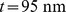 for 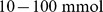 salt buffer [9], [32]. According to the size 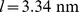 of the unit cylinder we find 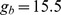 and 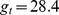.

### Langevin dynamics and global thermostat implementation

Although well adapted to mechanical simulations, ODE lacks coupling to a thermal bath. As physics engines impose to deal with dynamics equations including inertial terms, in particular for computing constraint forces (collected in 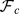), we need to turn to some implementation of stochastic isothermal molecular dynamics in order to thermalize the system: *isothermal* to simulate the system at constant temperature, *stochastic* to ensure ergodicity. The corresponding algorithms are all related to Langevin dynamics and can be cast into local and global thermostats. In local thermostats, such as standard Langevin dynamics, a correction force including both a frictional term and a stochastic term is exerted on each particle to drive the system to the canonical distribution at a prescribed temperature. Global schemes of Langevin dynamics are designed to minimize the perturbation introduced by the thermostat on the Hamiltonian trajectory (so called “disturbance” as defined originally in [36]), hence on the dynamical properties, such as autocorrelation functions, and related quantities, such as diffusion coefficients. In these globally applied thermostats the stochastic term of the correction force acting on each particle is proportional to the momentum of that particle. Two main global algorithms have been designed so far: (a) Stochastic Velocity Rescaling methods, most notably the “global thermostat” introduced by Bussi and Parrinello [19], (b) the Nosé-Hoover Langevin thermostat [37]. Here we first show how to implement Langevin-Euler equation in the ODE software. Moreover we show that the global thermostat introduced by Bussi and Parrinello is so remarkably adapted to this implementation that it improves quite significantly the sampling efficiency with respect to local Langevin dynamics (by two orders of magnitude in typical situations), while preserving the time-dependent properties such as autocorrelation functions. The sampling efficiency is defined as usual as the number of independent configurations generated during the time necessary to reach thermal equilibrium.

To begin with, we add to the “mechanical” forces 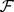 an additional, thermal contribution 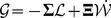 containing a frictional term 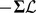 and a random force vector 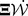. 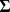 is the matrix of the coupling frequencies to the thermostat, 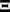 the matrix of white noise amplitudes and 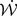 a generalized vector of normalized and independent Wiener processes. 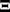 and 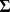 are related through the fluctuation-dissipation theorem, which reads here
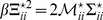
(31)where 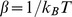 with 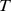 the temperature of the thermal bath and where the superscript 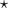 denotes that the matrices 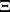 and 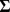 are chosen to be diagonal in the principal axis body frame (where the matrix 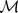 is diagonal by definition). For simplicity, we choose to fix all the 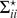 to a common frequency 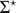. Note that 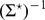 is the relaxation time of the thermostat, i.e. the autocorrelation time of the kinetic energy (see supplementary Figure S1).

We then improve the sampling efficiency of this Langevin dynamics by extending the “global thermostat” algorithm designed by Bussi and Parinello in 2008 [10] to physics engines. This algorithm allows faster yet correct sampling of the phase space in the canonical ensemble. However, it is designed for the translational degrees of freedom only. In order to apply it to an articulated rigid body system, we therefore have to extend it to the rotational degrees of freedom and adapt it to the ODE software. To this aim, we replace the traditional Langevin-Euler correction force (local thermostat) 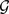 by a corresponding global version 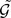, which reads
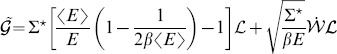
(32)
Eq.(32) shows that 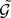 is proportional to 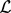, so that the stochastic force and torque globally associated with the thermostat is in the same direction as 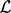. Hence, a free particle, i.e. a particle not connected to any other particle, will move on a straight line between two collisions. Note that, nevertheless, the particle will undergo Brownian motion along this straight trajectory. The global version of the Langevin dynamics minimizes the disturbance induced by the thermostat on the Hamiltonian trajectory (equal to 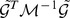 according to its definition in Ref. [19], but extended here to the rotational degrees of freedom), nevertheless retaining the same thermalization speed as usual Langevin dynamics (see supplementary Figure S1).

**Figure 2:**
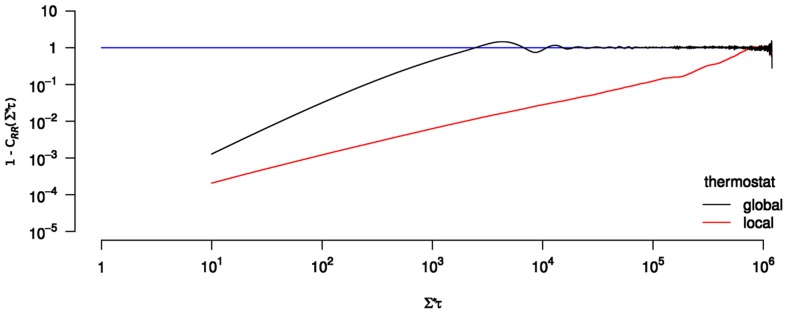
Global thermostat efficiency test. Complementary autocorrelation function 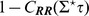 of the end-to-end distance of a DNA molecule simulated with the global Langevin thermostat (black) compared with the same function simulated with the local Langevin thermostat (red). 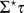 is the dimensionless lag-time with 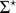 the thermostat coupling frequency. The DNA molecule is composed of 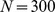 rigid cylinders of radius 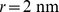 and 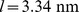. The other parameters of the simulation are given in Table 1. Cylinders are connected by ball-and-socket joints. The chain ends are free to diffuse here.

When used in the framework of a velocity-based algorithm such as ODE, the global thermostat presents a remarkable advantage. This is because, in this case the global Langevin contribution 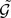 is decoupled from the constraint forces, in the sense that it cancels out in the equations for 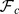. More precisely, with our definition of 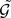 (see Eq.(32)), the contribution 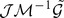 to the term 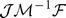 in Eq.(4) is always zero. In other words, 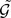 not only minimizes the disturbance of the Hamiltonian trajectory 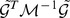, but also does not disturb the generalized constraint force 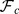. Both effects cooperate to achieve a dramatic acceleration of the simulation sampling, that is, in the case of our model, approximately 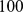 times faster than with the local thermostat (see below “Preliminary tests of validity and performance of the global thermostat” and Figure 2). Importantly this acceleration is compatible with the correct computation of dynamical properties, such as autocorrelation functions.

### Parameter settings of our implementation of ODE

In all DNA simulations presented in this article, we choose the length of the cylinders as the unit length 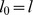, the mass of the cylinders as the unit mass 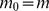 and the unit of thermal agitation 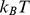 as the unit of energy 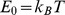, from which we deduce the unit of time 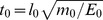. The complete set of parameters of our simulations is given in Table 1. We also choose to deal collisions with a restitution coefficient equal to 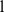 without surface friction. Hence, when two rigid bodies collide, the constraint force 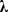 imposed by the contact joint that temporarily connects them is directed along their common normal 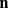 at the contact point. We finally choose an error reduction parameter 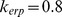 and a constraint force mixing parameter 
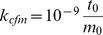
.

**Table 1:** Main set of parameters used for the DNA model and for the numerical simulations.

Entity	Parameter	Typical value	Definition
DNA parameters	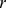	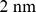	DNA cylinder effective radius in 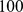 mmol salt
		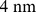	DNA cylinder effective radius in 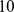 mmol salt
	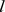	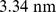	DNA cylinders length (10 basepairs)
	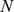	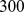	number of DNA cylinders
			(corresp. to a contour length 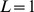 µm)
	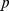	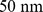	bending persistence length in 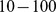 mmol salt
	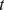	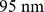	twisting persistence length in 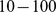 mmol salt
	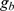	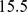	bending rigidity constant
			(corresp. to bending persistence length : 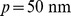 )
	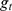	28.4	twisting rigidity constant
			(corresp. to twisting persistence length : 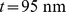 )
	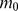	1.162 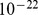 kg	mass of each DNA cylinder (10 basepairs)
Time units	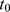	1.7660 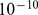 s	natural system time unit 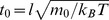
Simulation parameters	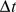	0.000592 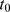	simulation time step
	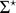	10 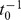	thermostat coupling frequency
	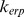	0.8	ODE error reduction parameter
	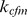	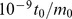	ODE constraint force mixing parameter (hard)
	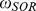	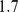	SOR relaxation factor
	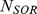	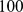	number of successive-over-relaxations

## Results/Discussion

### Preliminary tests of validity and performance of the global thermostat

We start to validate our methodology by simulating a DNA molecule without any constraint applied on the bead (neither stretching nor twisting). To this aim, we first check the equipartition theorem. When the system is at thermal equilibrium, its temperature is related to the kinetic energy through the equation
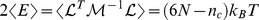
(33)where 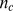 is the number of non-redundant holonomic constraints. This relation is standard since 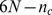 is just the number of degrees of freedom (dof) of the system. Moreover the distribution of the kinetic energy of the system at thermal equilibrium follows a Boltzmann law and therefore reads:
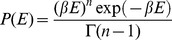
(34)with 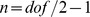. We checked this relation for a DNA molecule of length 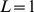 µm coupled to the two different Langevin thermostats, local and global respectively. The resulting histograms are shown in Figure 1, confirming that: (i) the kinetic energy is correctly sampled at thermal equilibrium with both thermostats, (ii) there is indeed no energy stored in the joints, although these have been softened by effective springs (see above the error reduction parameter 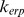 in subsection “Introduction to physics engines”).

We then quantified the simulation sampling efficiency by means of the autocorrelation function 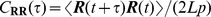 of the end-to-end distance 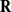 of a DNA molecule of length 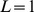
µm coupled to the two different Langevin thermostats, local and global respectively. Here the average is performed over the time 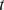 and 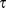 denotes the lag-time of the autocorrelation function. A demonstration of the performance of the global thermostat in terms of relaxation rapidity is given in Figure 2. Fitting the exponential decrease of both relaxation curves shows that with use of the global thermostat we reach the saturation value at 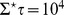 whereas this value is reached at 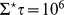 in the case of the local thermostat, thus resulting in an acceleration factor of about 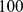 for this system composed of 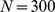 articulated rigid bodies. Note that the dimensionless lag-time 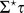 is equal to 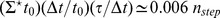 (see Table 1), with 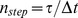 the corresponding number of time steps. Then a typical run using the global thermostat is of the order of tens of millions of time steps, whereas it is of the order of billions of time steps with usual Langevin dynamics. A striking illustration of the sampling acceleration provided by the global thermostat is also given in supplementary Video S1.

We also compute the tangent-tangent correlation function 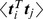 along the polymer with both local and global thermostats. No significant deviations were found between both thermostats. Results obtained with the global thermostat are plotted in supplementary Figure S2 along with the corresponding theoretical curves.

A simple calculation shows that the tangent-tangent correlation function decreases as 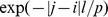, from which one can calculate the average bending 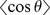. With the DNA persistence length 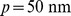, this quantity amounts to 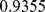, to be compared to the result from a fit of the simulation curves, giving 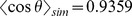. The same comparison can be done for the twist angle (with 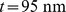), for which the simulation average 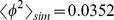 matches the theoretical value 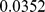. These comparisons show that our simulation results are in very good agreement with the analytical formulae, thus validating (i) our implementation of the bending and twisting rigidities and (ii) the correct sampling of the DNA conformation space by means of the global Langevin thermostat.

### Comparison with the experimental DNA stretching response

**Figure 3:**
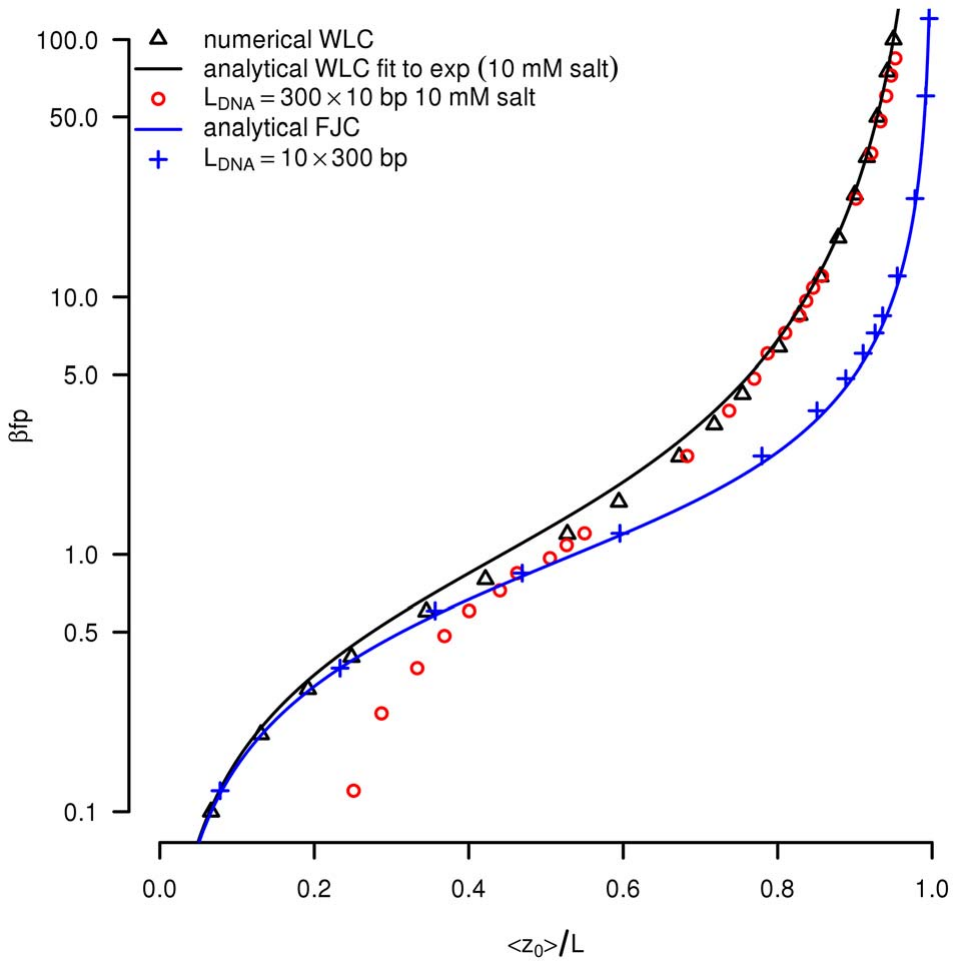
Comparison to force-extension curves. Red circles: dimensionless stretching force 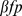 as a function of the mean relative extension 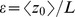. Here the radius of the cylinders is 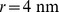 corresponding to the 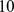
mmol monovalent salt buffer used in [1]. The other parameters of the simulation are given in Table 1. For comparison, the black solid line reproduces the analytical Worm-Like-Chain force-extension approximation formula [35]. Black triangles correspond to a numerical fit of the exact Worm-Like-Chain model [34] with the same persistence length 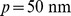. Blue crosses: we also show simulations in the limit case 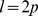, with no torsional rigidity (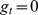) and no collisions, and compare it to the theoretical force-extension curve of a Freely-Jointed-Chain (FJC, blue solid line). The statistical error bars on the simulation points are all smaller than the symbol size.

We then simulate reference force-extension curves, both theoretical and experimental. We thus perform simulations at given stretching force 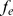 along the z-axis (normal to the DNA anchor surface), and without torsional constraints on the magnetic bead. In order to fit the experimental data obtained by Smith et al [1] in 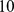 monovalent salt buffer, we set here the DNA radius (i.e. the radius of the unit cylinders) to 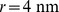. The resulting force-extension curve is given in Figure 3 where we plot (red circles) the dimensionless stretching force 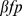 as a function of the dimensionless mean relative extension 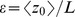. Here 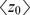 denotes the mean DNA extension, i.e. the mean distance between the bead and the anchor surface, at zero torsional constraint. For comparison, we also plot (black solid line) the analytical Worm-Like-Chain (WLC) interpolation fitting curve proposed by Bouchiat et al [35] as well as the numerical solution of the WLC model (black triangles) obtained by Marko and Siggia with the same persistence length 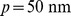 [34]. The simulation reproduces pretty well the WLC behavior, thus validating our implementation of the DNA bending rigidity. Note that, at low forces, the extension saturates at a value greater than zero because of the impenetrable ground and magnetic bead that both confine the DNA molecule. This effect is more pronounced than in the experimental curve [1] because the ratio of the bead radius to the DNA length is higher in our simulations.

In Figure 3, we also show the results obtained in the limit case 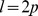, for which 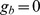 (see Eqs.(24–25)), and when there are no collisions. In this case we expect to observe a Freely-Jointed-Chain response (with 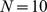 segments of 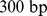 each). The analytical force-extension relation for FJC is given by the well-known expression as a Langevin function 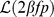 and it is also reproduced in Figure 3. Again, the simulation results are in very good agreement with the theoretical formula.

### Comparison with the experimental DNA torsional response

More interestingly, magnetic tweezers also allow the application of a torsional strain on a single DNA molecule at constant stretching force. This torsional strain is equal to the number of turns of the magnetic bead around the z-axis due to the rotation of the magnets. The number of turns of the bead is also equal to 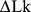, the variation of the linking number of the DNA double helix with respect to the intrinsic twist of the DNA double helix 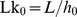 with 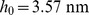 the pitch of the DNA. And we define as usual the DNA relative overtwist as 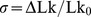.

#### Simulations at constant strain (fixed overtwist)

**Figure 4:**
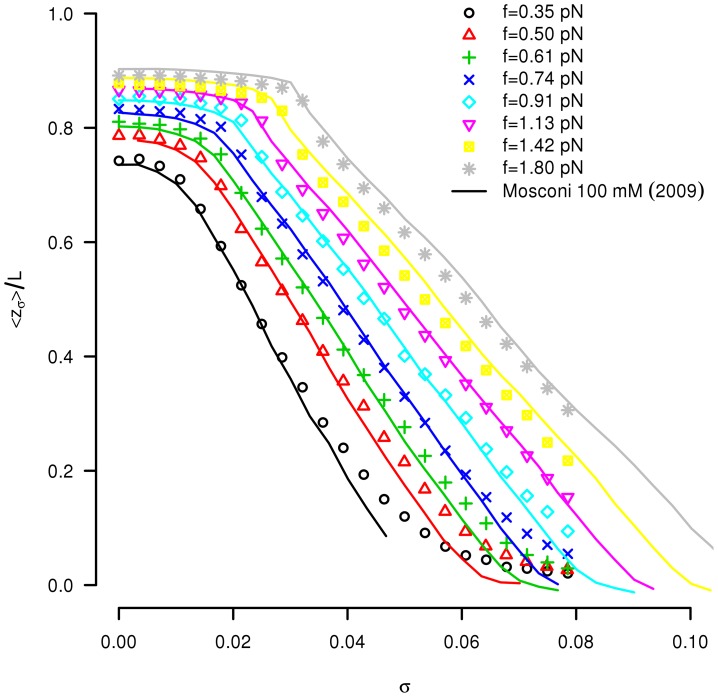
Comparison to experimental extension-rotation curves. Mean relative extension 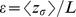 as a function of the imposed overtwist 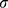 for different stretching forces 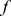 in the range of 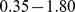 pN. We superimpose on to the simulation results (symbols) the experimental results from [32] (lines). The statistical error bars on the simulation points are smaller than the symbol size.

We simulate the rotation-extension behaviour of our DNA model by imposing the bead rotation and compare to experimental data from [32], where the mean relative extension 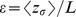 is given for different stretching forces as a function of the fixed relative overtwist 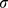 (Figure 4). An example of the simulated dynamics at a stretching force 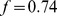
pN and 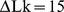 is shown in supplementary Video S2. Again excellent agreement is observed between the experimental bell-shape curves (also called “hat curves”) and the simulated curves. We went further to check the validity of the DNA radius which is set here to 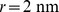 according to the 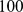 monovalent salt buffer used in these experiments. To this aim we refer to a series of papers by Neukirch and co–workers [9], [24], [25] where they showed that the linear part of the “hat curves” can be expressed as a function of the supercoiling radius 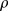, the superhelical angle 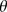 and the ratio 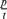 of the twisting and bending persistence lengths, as
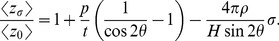
(35)where 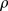 and 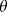 depend on the force applied on the bead.

The comparison between the experimental estimations of the three parameters 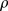, 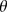, and 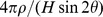 deduced from [32] and the corresponding results from our simulations are given as functions of the applied force in the three supplementary Figures S3, S4, S5. The good agreement observed validates the value applied to the effective DNA radius 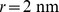 in 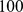
mmol monovalent salt buffer.

#### Simulations at constant stress (fixed torque)

In the characteristic rotation-extension “hat curves” the DNA extension decreases linearly as a function of 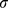 once it reaches a critical point where the resulting applied torque crosses a critical buckling value 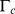. This linear decrease corresponds to the formation of plectonemes: any additional turn beyond the buckling transition is absorbed into the growing plectoneme without changes in the torque [10]. As standard magnetic tweezers cannot measure torques, the buckling torque can be only indirectly deduced by integrating the change of the molecule extension with respect to the applied force [32], [38]. However, new set ups have recently been proposed that enforce a torque and allow its measurement: one of them uses an angular optical trap [39]–[41], another one a magnetic nanorod coupled to a magnetic bead [42] and a third one a soft magnetic tweezer [43]. These innovative experiments confirm that the torque stays constant during the plectoneme formation, and allow the investigation of the dependence of 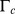 on the applied stretching force.

**Figure 5:**
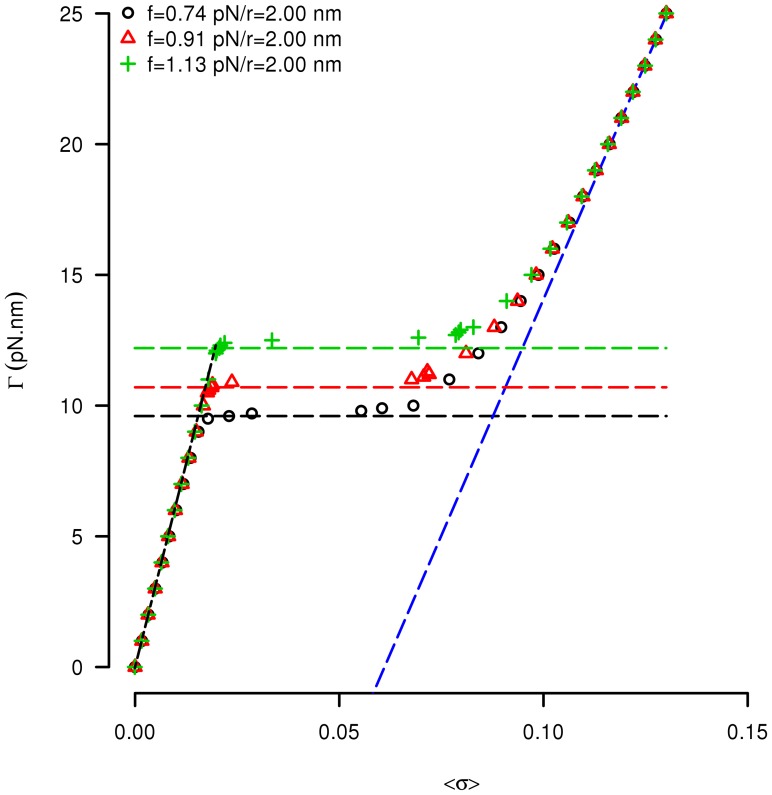
Torque computation. Twisting torque 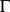 as a function of the average overtwist 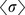 for stretching forces 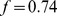, 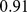 and 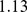 pN. Symbols are the simulation results. Horizontal lines show the values of the critical torques estimated from the experimental results at the corresponding stretching forces [32]. Oblique black dotted line: experimental results in the pure extended state (low 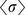). Oblique blue dotted line: asymptotic behavior of the twisting torque as a function of the simulated average overtwist in the pure plectonemic state (high 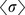): 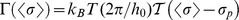 with 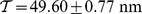 and 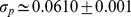; 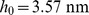 is the pitch of the DNA double helix.

Our technique also allows us to simulate the DNA response when both the stretching force 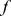 and the twisting torque 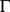 are imposed, while the number of turns of the magnetic bead 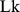 is free to evolve. In this case we compute the average overtwist 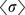 for fixed values of the twisting torque 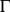. Figure 5 shows 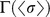 for three different values of the external force. A clear transition from “pure extended” DNA (left part of the curves) to “pure plectonemic” DNA (right part) is observed at (almost) constant critical torque 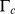. In the pure extended state, simulations and experimental results [32] are in very good agreement. In this regime 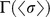 is linear and the corresponding slope agrees with theoretical predictions [10]. The estimations of the critical torque obtained in [32] by integrating the change in the molecule extension are reported as horizontal lines in Figure 5. Our simulation correctly reproduces these values, including the dependence of 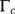 on the stretching force 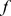 throughout the whole range of salt concentrations explored (data not shown).

Note that we sporadically obtained two plectonemes in the same DNA molecule during some simulation runs. Moreover we also observed that plectonemes diffuse along the DNA molecule when the global thermostat is started up (see supplementary Video S1). Interestingly, both features - multiplectonemes and plectoneme diffusion - have been recently observed experimentally [44] and theoretically explained [45]. Note that nevertheless these observations have been obtained with a 21-kb DNA molecule, hence with a much longer molecule than in our simulations (3-kb). This may explain why we did not observe the “multiplectoneme phase” described in ([45]).

**Figure 6:**
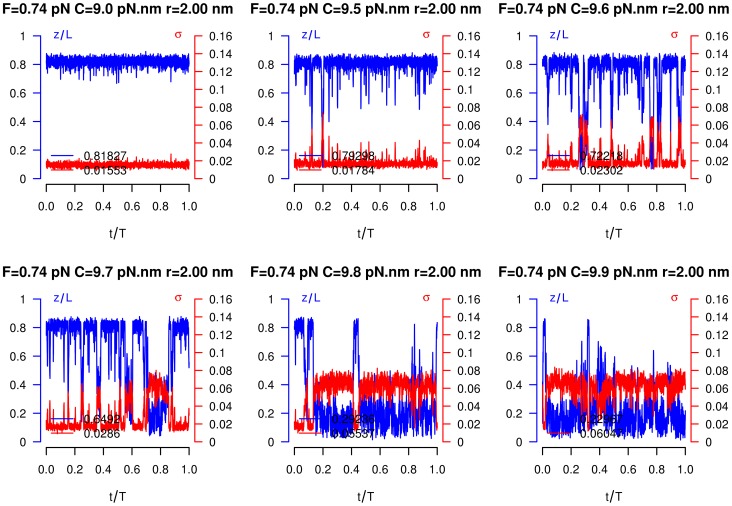
Buckling behavior. Buckling instability with stretching force of 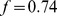 pN and six different torques 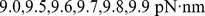. (Blue) DNA relative extension. (Red) DNA overtwist. DNA relative extension and overtwist are monitored as a function of the number of simulation steps. All these recordings have been obtained after thermal equilibrium has been reached.

The increase of the average overtwist 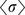 observed *at* the critical torque in Figure 5 corresponds to the formation of plectonemes, until the entire DNA molecule is supercoiled. We explored this critical regime by recording, at given 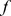 and 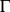, the time evolution of both the molecule length and overtwist. When the critical torque is approached, a clear buckling instability appears, with DNA fluctuating between the pure extended state, characterized by a small supercoiling and a large extension, and a pure plectonemic state, with opposite characteristics [10] (Figure 6). Note the anticorrelation between supercoiling and extension.

Beyond the buckling torque, DNA is in the pure plectonemic state, and an increasing torque will tighten the molecule supercoiling. Interestingly, while experimental results [32] do not provide measurements of the average overtwist 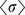 in this pure plectonemic state, our simulations allow to explore this regime. An example of the simulated dynamics at a stretching force 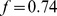
pN and twisting torque 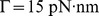 is shown in supplementary Video S3. We observe that our simulation results deviate from Marko's theoretical prediction according to which 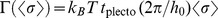, with 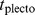 the twist persistence length of the plectoneme and 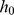 the pitch of the DNA double helix [10]. Instead we get an affine law which may be cast in a similar form: 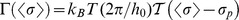 with 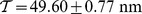 and 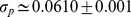. The presence of the reference value 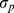 seems rather natural since this is the overtwist above which the system enters the pure plectonemic state. The meaning of the 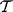 length is an open question, but we notice that its value is close to the DNA bending persistence length 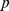.

### Concluding remarks

The method developed here can conveniently describe all physiological situations involving DNA positive supercoiling, which are of main importance for DNA transcription or replication. One limitation of the modeling developed so far is that extensive modifications of the double helix structure are not accounted for, e.g. base pair opening that occurs when negative supercoiling is applied (DNA denaturation), or the S-DNA transition observed at extremely high force, or the P-DNA transition under very positive torque. Nevertheless this methodology gives an unparalleled opportunity to study more complex biological systems, such as protein-DNA complexes: in particular, we are currently addressing the modeling of magnetic tweezers response of chromatin fibres [7], by associating our *in silico* DNA model with a rigid body representation of the histone octamer. More recently, *in vivo* experiments also enable the measurement of the dynamics of chromosome *loci* in the cell nucleus [11], [12], [46].

## Supporting Information

Figure S1Autocorrelation function of the kinetic energy 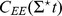 (symbols) of a polymer of size 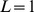 µm and persistence length 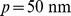 and for three different cylinder lengths: 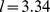 (circles), 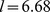 (triangles) and 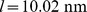 (crosses). We compare the theoretical exponential model 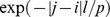 for the tangent-tangent correlation to our simulation results (blue lines).(TIF)

Figure S2Average tangent-tangent correlation 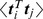 (symbols) for a polymer of size 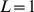 µm and persistence length 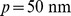 and for three different cylinder lengths: 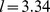 (circles), 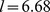 (triangles) and 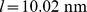 (crosses). We compare the theoretical exponential model 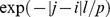 for the tangent-tangent correlation to our simulation results (blue lines).(TIFF)

Figure S3Supercoiling radius 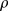 estimation from simulation results (black circles) and from experimental results [32] (red triangles).(TIFF)

Figure S4Helical angle estimation 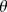 from simulation results (black circles) and from experimental results [32] (red triangles).(TIFF)

Figure S5Slope estimation 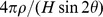 from simulation results (black circles) and from experimental results [32] (red triangles).(TIFF)

Text S1 In this appendix we address the problem of how to obtain a correct and computationally efficient definition of the bending and twisting rigidities for a polymer in general and a DNA molecule in particular.
(PDF)

Video S1Illustration of the sampling efficiency of global versus local thermostat. This 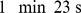 video records the simulated dynamics of a DNA molecule manipulated by magnetic tweezers after thermal equilibrium has been reached. The stretching force is 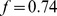
pN and the number of turns of the bead is fixed to 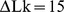. From 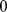 to 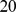
s the simulation is run with Langevin local thermostat. Then the global thermostat is started up at 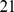
s and run until 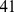
s, strikingly accelerating the dynamics of the DNA molecule. Moreover the plectoneme starts to diffuse along the DNA molecule. Then the local thermostat is run again from 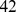
s to 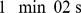, resulting in a dramatic slowing down of the dynamics. And finally the global thermostat is started up again at 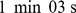 and run until the end of the video at 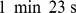. The DNA molecule is composed of 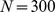 rigid cylinders of radius 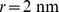 and 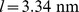. The other parameters of the simulation are given in Table 1. This video is also available at http://vimeo.com/51918121.(AVI)

Video S2Simulated dynamics of a DNA molecule manipulated by magnetic tweezers with a stretching force 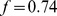
pN and fixed number of turns of the bead 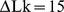. This dynamics is performed with the global thermostat after thermal equilibrium has been reached. Note the diffusion of the plectoneme all along the DNA molecule. The DNA molecule is composed of 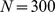 rigid cylinders of radius 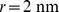 and 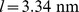. The other parameters of the simulation are given in Table 1. This video is also available at http://vimeo.com/51918378.(AVI)

Video S3Simulated dynamics of a DNA molecule manipulated by magnetic tweezers with a stretching force 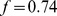
pN and fixed twisting torque 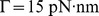. Global thermostat is used all over the simulation. At initial time the number of turns of the bead is zero and the torque starts to be applied to the bead (the rotation of the bead can be followed thanks to the small orange tag on the sphere). Because the applied torque (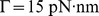) is well above the buckling torque (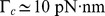), the DNA molecule is in the pure plectonemic state at the end of the simulation. The DNA molecule is composed of 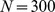 rigid cylinders of radius 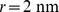 and 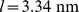. The other parameters of the simulation are given in Table 1. This video is also available at http://vimeo.com/51918151.(AVI)

## References

[1] Smith S, Finzi L, Bustamante C (1992) Direct mechanical measurements of the elasticity of single dna molecules by using magnetic beads. Science 258: 1122–1126. 1439819 10.1126/science.1439819

[2] Strick TR, Allemand JF, Bensimon D, Bensimon A, Croquette V (1996) The elasticity of a single supercoiled dna molecule. Science 271: 1835–1837. 8596951 10.1126/science.271.5257.1835

[3] Bryant Z, Stone MD, Gore J, Smith SB, Cozzarelli NR, et al (2003) Structural transitions and elasticity from torque measurements on dna. Nature 424: 338–341. 12867987 10.1038/nature01810

[4] Strick TR, Croquette V, Bensimon D (2000) Single-molecule analysis of DNA uncoiling by a type II topoisomerase. Nature 404: 901–904. 10786800 10.1038/35009144

[5] Koster DA, Croquette V, Dekker C, Shuman S, Dekker NH (2005) Friction and torque govern the relaxation of DNA supercoils by eukaryotic topoisomerase IB. Nature 434: 671–674. 15800630 10.1038/nature03395

[6] Bancaud A, Conde e Silva N, Barbi M, Wagner G, Allemand JF, et al (2006/05//print) Structural plasticity of single chromatin fibers revealed by torsional manipulation. Nat Struct Mol Biol 13: 444–450. 16622406 10.1038/nsmb1087

[7] Bancaud A, Wagner G, Conde e Silva N, Lavelle C, Wong H, et al (2007/07/06) Nucleosome chiral transition under positive torsional stress in single chromatin fibers. Molecular cell 27: 135–147. 17612496 10.1016/j.molcel.2007.05.037

[8] Moroz JD, Nelson P (1997) Torsional directed walks, entropic elasticity, and dna twist stiffness. Proc Natl Acad Sci USA 94: 14418. 9405627 10.1073/pnas.94.26.14418 PMC25005

[9] Neukirch S (2004) Extracting dna twist rigidity from experimental supercoiling data. Phys Rev Lett 93: 198107. 15600890 10.1103/PhysRevLett.93.198107

[10] Marko JF (2007) Torque and dynamics of linking number relaxation in stretched supercoiled dna. Phys Rev E 76: 021926. 10.1103/PhysRevE.76.021926 17930084

[11] Heun P, Laroche T, Shimada K, Furrer P, Gasser SM (2001) Chromosome dynamics in the yeast interphase nucleus. Science 2181–2186. 11739961 10.1126/science.1065366

[12] Albert B, Mathon J, Shukla A, Saad H, Normand C, et al (2013) Systematic characterization of the conformation and dynamics of budding yeast chromosome XII. J Cell Biol 202: 201–210. 23878273 10.1083/jcb.201208186 PMC3718979

[13] Vologodskii AV, Levene SD, Klenin KV, Frank-Kamenetskii M, Cozzarelli NR (1992) Conformational and thermodynamic properties of supercoiled dna. Journal of Molecular Biology 227: 1224–1243. 1433295 10.1016/0022-2836(92)90533-p

[14] Vologodskii A (1994) Dna extension under the action of an external force. Macromolecules 27: 5623–5625.

[15] Ryckaert JP, Ciccotti G, Berendsen HJ (1977) Numerical integration of the cartesian equations of motion of a system with constraints: molecular dynamics of n-alkanes. Journal of Computational Physics 23: 327–341.

[16] van Gunsteren W, Berendsen H, Rullmann J (1981) Stochastic dynamics for molecules with constraints. Molecular Physics 44: 69–95.

[17] Erleben K (2005) Stable, Robust, and Versatile Multibody Dynamics Animation. Ph.D. thesis, The Department of Computer Science, University of Copenhagen, Denmark.

[18] Smith R (2008). Open dynamic engine. http://www.ode.org.

[19] Bussi G, Parrinello M (2008) Stochastic thermostats: comparison of local and global schemes. Computer Physics Communications 179: 26–29.

[20] Fiser D. Library for collision detection between complex shapes. http://libccd.danfis.cz.

[21] Grassia FS (1998) Practical parameterization of rotations using the exponential map. J Graph Tools 3: 29–48.

[22] Young DM (1950) Iterative methods for solving partial difference equations of elliptical type. Ph.D. thesis, Havard University.

[23] Cottle RW, Dantzig GB (1968) Complementary pivot theory of mathematical programming. Linear Algebra and its Applications 1: 103–125.

[24] Clauvelin N, Audoly B, Neukirch S (2008) Mechanical response of plectonemic dna: An analytical solution. Macromolecules 41: 4479–4483.

[25] Clauvelin N, Audoly B, Neukirch S (2009) Elasticity and electrostatics of plectonemic dna. Biophysical Journal 96: 3716–3723. 19413977 10.1016/j.bpj.2009.02.032 PMC2711414

[26] Brutzer H, Luzzietti N, Klaue D, Seidel R (2010) Energetics at the dna supercoiling transition. Biophysical Journal 98: 1267–1276. 20371326 10.1016/j.bpj.2009.12.4292 PMC2849096

[27] Argudo D, Purohit PK (2012) The dependence of dna supercoiling on solution electrostatics. Acta Biomaterialia 8: 2133–2143. 22330280 10.1016/j.actbio.2012.01.030

[28] Stigter D (1977) Interactions of highly charged colloidal cylinders with applications to doublestranded dna. Biopolymers 16: 1435–1448. 880366 10.1002/bip.1977.360160705

[29] Brian AA, Frisch HL, Lerman LS (1981) Thermodynamics and equilibrium sedimentation analysis of the close approach of dna molecules and a molecular ordering transition. Biopolymers 20: 1305–1328. 7284570 10.1002/bip.1981.360200615

[30] Rybenkov VV, Cozzarelli NR, Vologodskii AV (1993) Probability of dna knotting and the effective diameter of the dna double helix. Proc Natl Acad Sci USA 90: 5307–5311. 8506378 10.1073/pnas.90.11.5307 PMC46705

[31] Rybenkov VV, Vologodskii AV, Cozzarelli NR (1997) The effect of ionic conditions on dna helical repeat, effective diameter and free energy of supercoiling. Nucleic Acids Research 25: 1412–1418. 9060437 10.1093/nar/25.7.1412 PMC146597

[32] Mosconi F, Allemand JF, Bensimon D, Croquette V (2009) Measurement of the torque on a single stretched and twisted dna using magnetic tweezers. Phys Rev Lett 102: 078301. 19257716 10.1103/PhysRevLett.102.078301

[33] Hagerman PJ (1988) Flexibility of dna. Annual Review of Biophysics and Biophysical Chemistry 17: 265–286. 10.1146/annurev.bb.17.060188.001405 3293588

[34] Marko JF, Siggia E (1995) Stretching dna. Macromolecules 28: 8759–8770.

[35] Bouchiat C, Wang M, Allemand JF, Strick T, Block S, et al (1999) Estimating the persistence length of a worm-like chain molecule from force-extension measurements. Biophysical Journal 76: 409–413. 9876152 10.1016/s0006-3495(99)77207-3 PMC1302529

[36] Berendsen HJC, Postma JPM, van Gunsteren WF, DiNola A, Haak JR (1984) Molecular dynamics with coupling to an external bath. The Journal of Chemical Physics 81: 3684–3690.

[37] Samoletov A, Chaplain M, Dettmann C (2007) Thermostats for slow configurational modes. Journal of Statistical Physics 128: 1321–1336.

[38] Zhang H, Marko JF (2008) Maxwell relations for single-dna experiments: Monitoring protein binding and double-helix torque with force-extension measurements. Phys Rev E 77: 031916. 10.1103/PhysRevE.77.031916 18517431

[39] Forth S, Deufel C, Sheinin MY, Daniels B, Sethna JP, et al (2008) Abrupt buckling transition observed during the plectoneme formation of individual dna molecules. Phys Rev Lett 100: 148301. 18518075 10.1103/PhysRevLett.100.148301 PMC3019760

[40] Christopher Deufel, Scott Forth, Simmons Chad R, Siavash Dejgosha, Wang Michelle D (2007) Nanofabricated quartz cylinders for angular trapping: DNA supercoiling torque detection. Nat Meth 4: 223–225. 10.1038/nmeth1013 17322891

[41] Wang M, Yin H, Landick R, Gelles J, Block S (1997) Stretching dna with optical tweezers. Biophysical Journal 72: 1335–1346. 9138579 10.1016/S0006-3495(97)78780-0 PMC1184516

[42] Celedon A, Nodelman IM, Wildt B, Dewan R, Searson P, et al (2009) Magnetic tweezers measurement of single molecule torque. Nano Letters 9: 1720–1725. 19301859 10.1021/nl900631w PMC4823137

[43] Mosconi F, Allemand JF, Croquette V (2011) Soft magnetic tweezers: A proof of principle. Rev Sci Instrum 82: 034302. 21456769 10.1063/1.3531959

[44] van Loenhout MTJ, de Grunt MV, Dekker C (2012) Dynamics of dna supercoils. Science 338: 94–97. 22983709 10.1126/science.1225810

[45] Emanuel M, Lanzani G, Schiessel H (2013) Multiplectoneme phase of double-stranded dna under tension. Phys Rev E 88: 022706. 10.1103/PhysRevE.88.022706 24032863

[46] Hajjoul H, Mathon J, Ranchon H, Goiffon I, Mozziconacci J, et al (2013) High throughput chromatin motion tracking in living yeast reveals the exibility of the fiber throughout the genome. Genome Research 23: 1829–1838. 24077391 10.1101/gr.157008.113 PMC3814883

