# Supplementary figures and images for: In Silico Single-Molecule Manipulation of DNA with Rigid Body Dynamics
Source: PLoS Comput Biol. 2014 Feb 20;10(2):e1003456. doi: 10.1371/journal.pcbi.1003456 (PMC3930497; doi:10.1371/journal.pcbi.1003456)

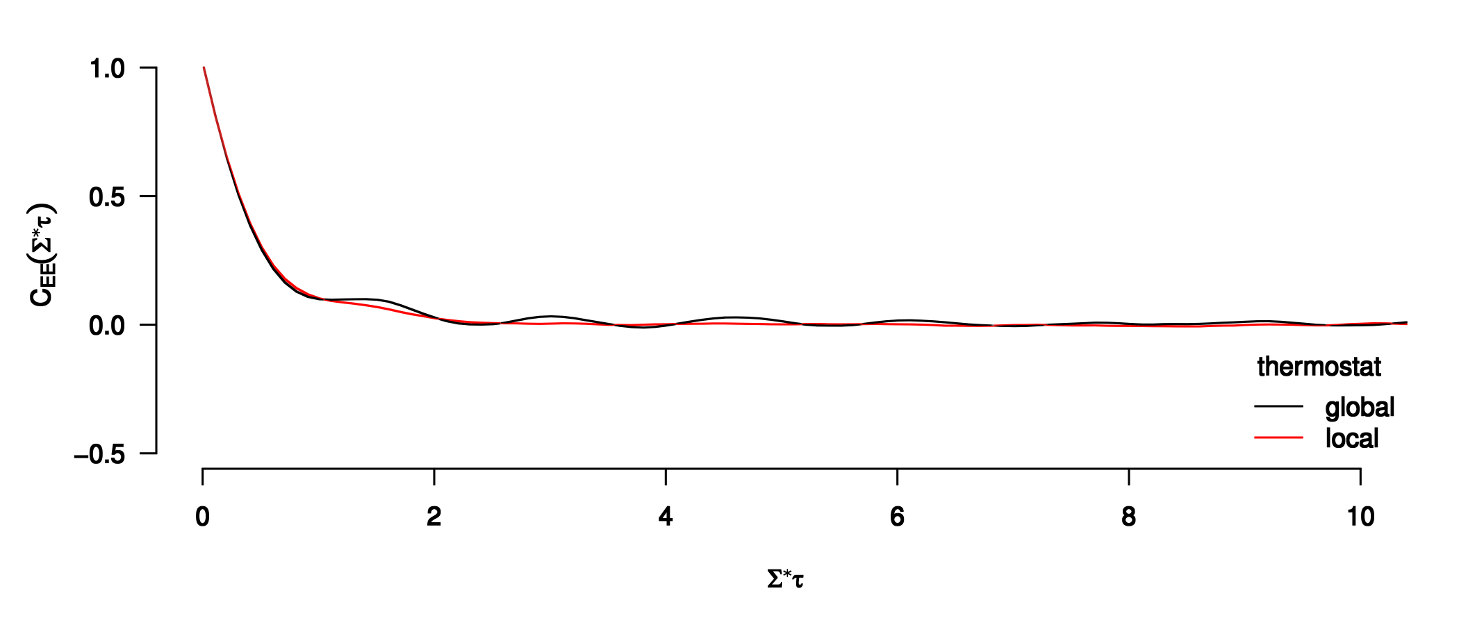

Supplement: Figure S1 — Autocorrelation function of the kinetic energy (symbols) of a polymer of size µm and persistence length and for three different cylinder lengths: (circles), (triangles) and (crosses). We compare the theoretical exponential model for the tangent-tangent correlation to our simulation results (blue lines). (TIF) [file pcbi.1003456.s001.tif]

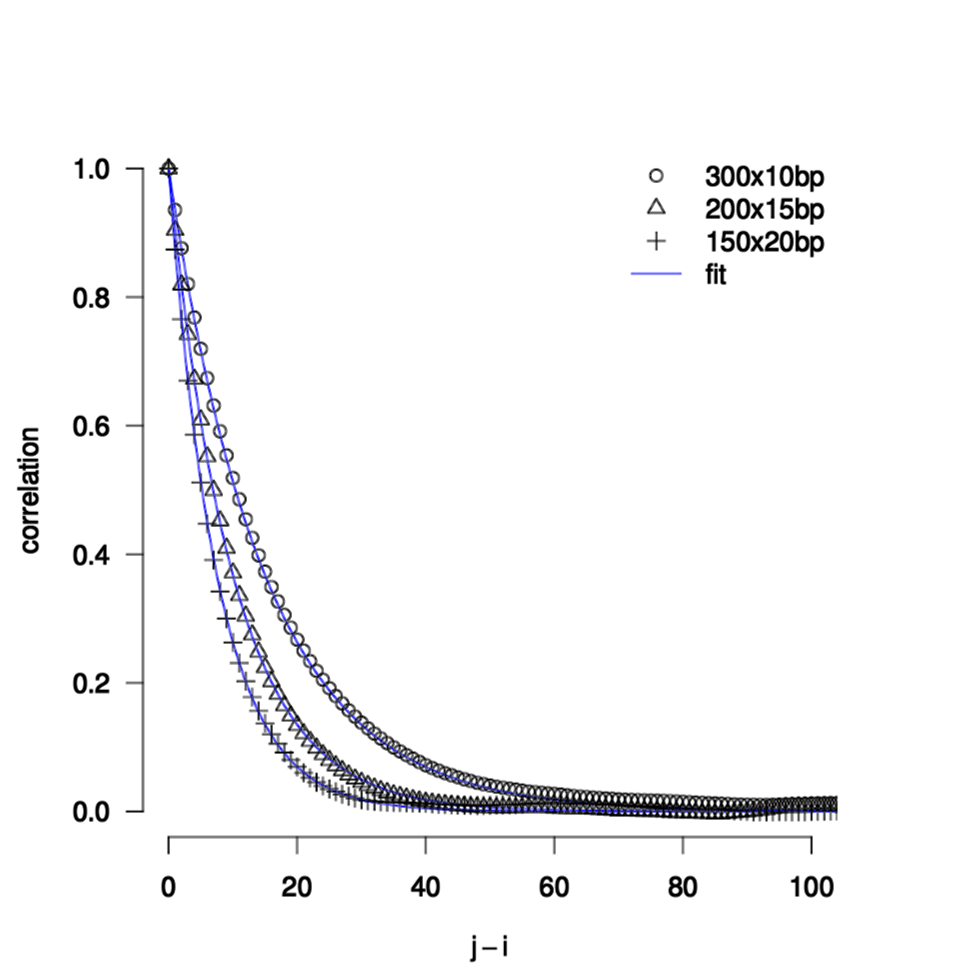

Supplement: Figure S2 — Average tangent-tangent correlation (symbols) for a polymer of size µm and persistence length and for three different cylinder lengths: (circles), (triangles) and (crosses). We compare the theoretical exponential model for the tangent-tangent correlation to our simulation results (blue lines). (TIFF) [file pcbi.1003456.s002.tiff]

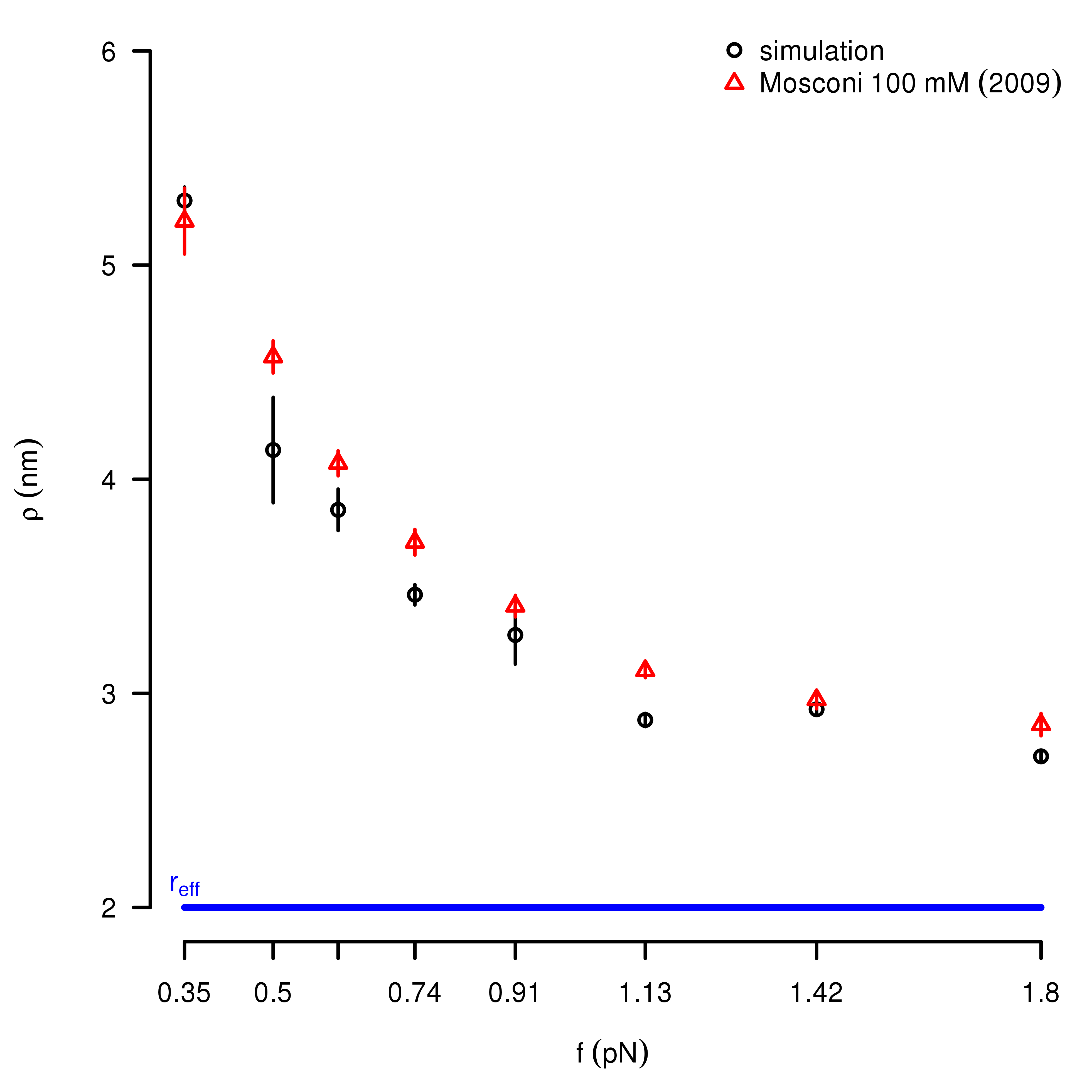

Supplement: Figure S3 — Supercoiling radius estimation from simulation results (black circles) and from experimental results [32] (red triangles). (TIFF) [file pcbi.1003456.s003.tiff]

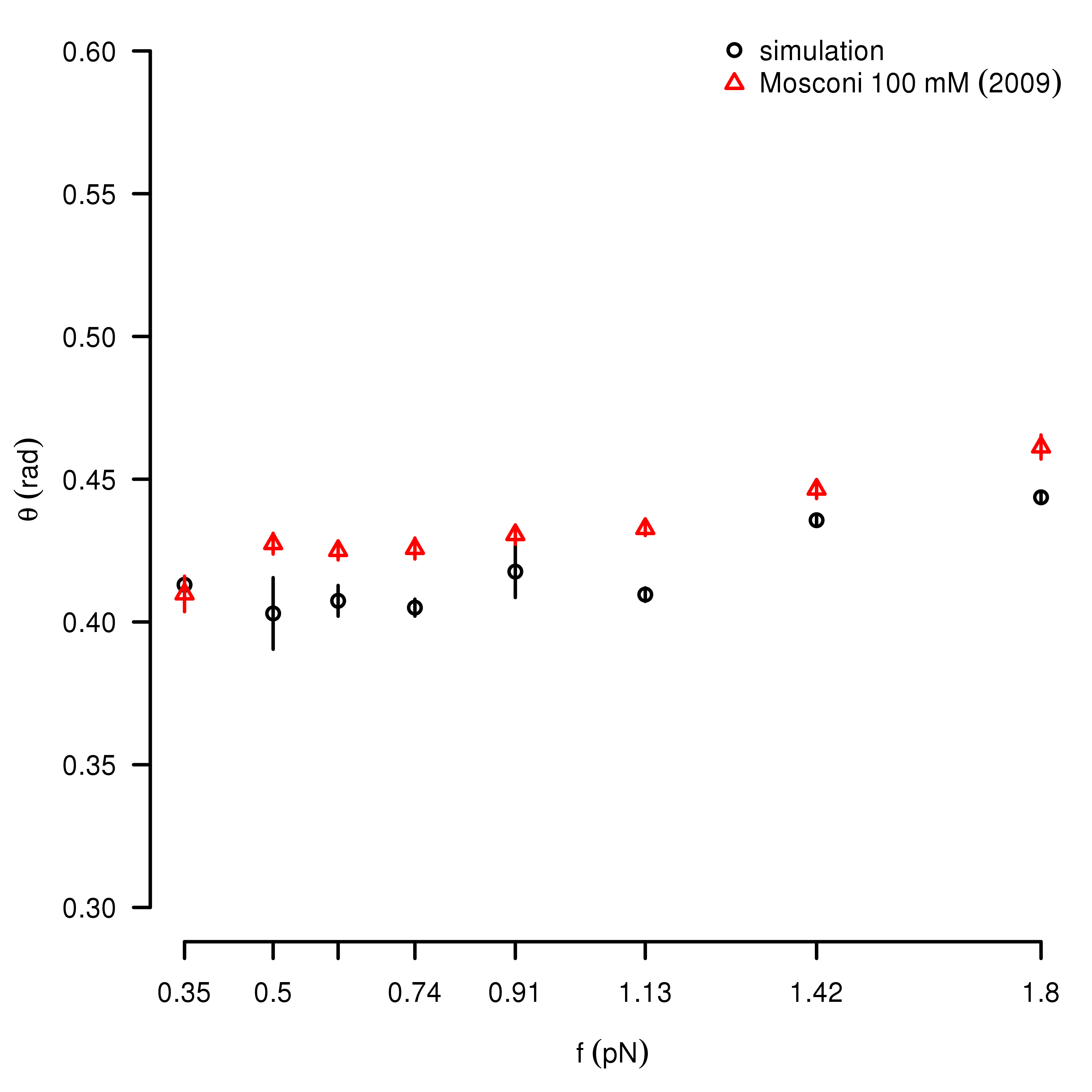

Supplement: Figure S4 — Helical angle estimation from simulation results (black circles) and from experimental results [32] (red triangles). (TIFF) [file pcbi.1003456.s004.tiff]

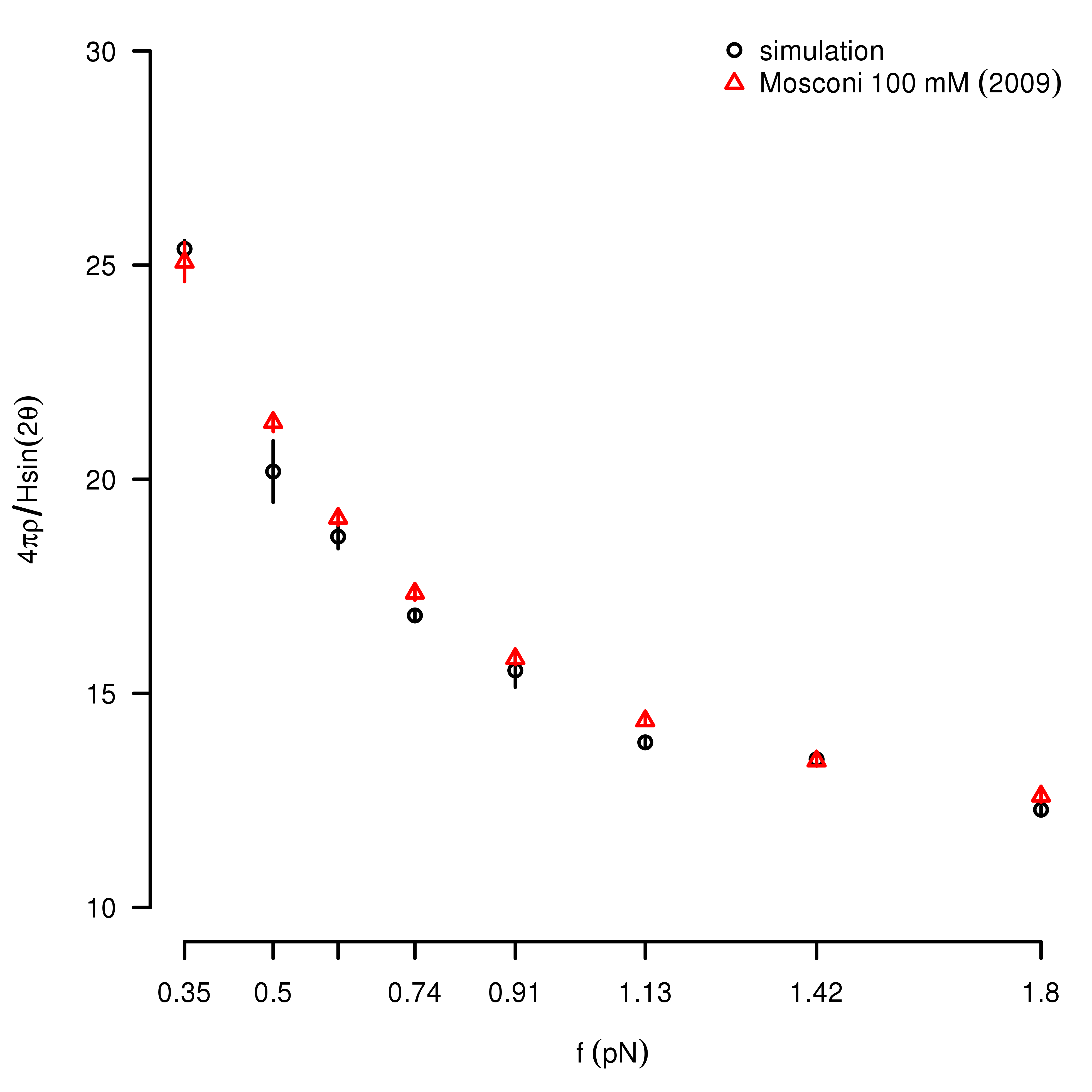

Supplement: Figure S5 — Slope estimation from simulation results (black circles) and from experimental results [32] (red triangles). (TIFF) [file pcbi.1003456.s005.tiff]
