# Supplementary material for: In Silico Single-Molecule Manipulation of DNA with Rigid Body Dynamics
Source: PLoS Comput Biol. 2014 Feb 20;10(2):e1003456. doi: 10.1371/journal.pcbi.1003456 (PMC3930497; doi:10.1371/journal.pcbi.1003456)
Supplement: Text S1 — In this appendix we address the problem of how to obtain a correct and computationally efficient definition of the bending and twisting rigidities for a polymer in general and a DNA molecule in particular. (PDF) [file pcbi.1003456.s006.pdf]

SUPPLEMENTARY SECTION
In silico single-molecule manipulation of DNA with rigid body dynamics: an efficient tool

P. Carrivain, M. Barbi, and J-M. Victor
Laboratoire de Physique Théorique de la Matière Condensée,
*Université Pierre et Marie Curie, 4, Place Jussieu, 75252 Paris Cedex 05, France**
(Dated: October 26, 2013)

SIMULATING THE BENDING AND TWISTING BEHAVIOUR OF A POLYMER

In this supplementary section we address the problem of how to obtain a correct definition of the bending and twisting rigidities for a polymer, hence for the case of a DNA molecule. We connect the cylinders to each other by ball-in-socket joints, i.e. freely rotating points linking the two bodies and needing three holonomic constraints. Let us define the material frame attached to a body by a tangent vector $\boldsymbol{t}$, a normal vector $\boldsymbol{m}$ and third vector $\widetilde{\boldsymbol{m}} = \boldsymbol{t} \otimes \boldsymbol{m}$. The two bodies connected by a ball-in-socket joint are implicitly connected by standard Euler transformation Z-X-Z (with the corresponding angles α, θ and ψ). The local chain bending is then defined by the cosine of bending angle

$$\cos\theta = \boldsymbol{t}_1^T \boldsymbol{t}_2 \tag{s1}$$

and the corresponding bend energy cost is of the form

$$\beta E_b = g_b \left(1 - \cos\theta\right) . \tag{s2}$$

The bending rigidity constant g_b in the previous equation is related to the polymer persistence length p, defined as the length over which the chain loses its orientation correlation. The relation between the constant g_b and the length p should be such that one recovers a Worm-Like-Chain behaviour as the limiting case $N \to \infty$. following Ref. [?], we thus equal the thermal average of the local chain bending, $\cos\theta$, to its ensemble average obtained from the square end-to-end distance. This equality reads:

$$\mathscr{L}\left(g_b\right) = \frac{k - l}{k + l} , \tag{s3}$$

where $\mathscr{L}$ is the Langevin function and k the Kuhn length, equal to twice the bending persistence length ($k = 2p$). The solution of this equation defines g_b and imposes that a Kuhn length corresponds to $p/2l$ rigid segments. We recall that, for DNA, estimates of k give 100 nm [? ? ? ?] for $10 - 500$ mmol salt buffer, which is the range of values used in this paper. The local polymer twist is defined as $\phi = \alpha + \psi$ or, in terms of unit vectors, by the equations

$$\left(1 + \boldsymbol{t}_1^T \boldsymbol{t}_2\right) \cos\phi = \boldsymbol{m}_2^T \boldsymbol{m}_1 + \widetilde{\boldsymbol{m}}_2^T \widetilde{\boldsymbol{m}}_1 \tag{s4}$$

$$\left(1 + \boldsymbol{t}_1^T \boldsymbol{t}_2\right) \sin\phi = \boldsymbol{m}_2^T \widetilde{\boldsymbol{m}}_1 - \widetilde{\boldsymbol{m}}_2^T \boldsymbol{m}_1 \tag{s5}$$

The twist energy then reads

$$\beta E_t = g_t \frac{\phi^2}{2} \tag{s6}$$

where the twist rigidity constant is defined similarly to its bending analogue as $g_t = t/l$, where t is the bending persistence length. Estimations for the DNA molecule give $t = 95$ nm in 10 mmol and 100 mmol salt buffer [? ?].

We now address the problem of defining bending and twist resisting torques in order to mimic the polymer rigidity. These definitions should be coherent with the previous expressions for the bending and twist energy and, since the energy only depends on the orientation of two successive segments, consistent definitions of resisting torques are

$$\boldsymbol{\Gamma}_b = -\left(\boldsymbol{t} \otimes \nabla_{\boldsymbol{t}} + \boldsymbol{m} \otimes \nabla_{\boldsymbol{m}} + \widetilde{\boldsymbol{m}} \otimes \nabla_{\widetilde{\boldsymbol{m}}}\right) E_b \tag{s7}$$

* carrivain@lptmc.jussieu.fr,victor@lptmc.jussieu.fr

and

$$\mathbf{\Gamma}_t = -\left(\boldsymbol{t} \otimes \nabla_{\boldsymbol{t}} + \boldsymbol{m} \otimes \nabla_{\boldsymbol{m}} + \widetilde{\boldsymbol{m}} \otimes \nabla_{\widetilde{\boldsymbol{m}}}\right) E_t \,. \tag{s8}$$

Moreover, one has to impose an action and reaction equality for any body pairs $\mathbf{\Gamma}^1_{b+t} = -\mathbf{\Gamma}^2_{b+t} = \mathbf{\Gamma}_{b+t}$.

Let us finally calculate explicitly the twist resisting torque $\mathbf{\Gamma}_t$. This derivation is aims to exemplify and clarify our procedure. We recall that giving the material frame relative to the first body, we can use standard Euler angles α, θ and ψ to find the orientation of the second body, as follows:

$$\begin{aligned}
\boldsymbol{m}_2 &= \left(\cos\psi\cos\alpha - \cos\theta\sin\alpha\sin\psi\right)\boldsymbol{m}_1 \\
&\quad + \left(\cos\psi\sin\alpha + \cos\theta\cos\alpha\sin\psi\right)\widetilde{\boldsymbol{m}}_1 \\
&\quad + \sin\psi\sin\theta\boldsymbol{t}_1 \\
\widetilde{\boldsymbol{m}}_2 &= \left(-\sin\psi\cos\alpha - \cos\theta\sin\alpha\cos\psi\right)\boldsymbol{m}_1 \\
&\quad + \left(-\sin\psi\sin\alpha + \cos\theta\cos\alpha\cos\psi\right)\widetilde{\boldsymbol{m}}_1 \\
&\quad + \cos\psi\sin\theta\boldsymbol{t}_1 \\
\boldsymbol{t}_2 &= \sin\theta\sin\alpha\boldsymbol{m}_1 - \sin\theta\cos\alpha\widetilde{\boldsymbol{m}}_1 + \cos\theta\boldsymbol{t}_1
\end{aligned} \tag{s9}$$

or inverse transformation as follows:

$$\begin{aligned}
\boldsymbol{m}_1 &= \left(\cos\psi\cos\alpha - \cos\theta\sin\alpha\sin\psi\right)\boldsymbol{m}_2 \\
&\quad - \left(\sin\psi\cos\alpha + \cos\theta\sin\alpha\cos\psi\right)\widetilde{\boldsymbol{m}}_2 \\
&\quad + \sin\alpha\sin\theta\boldsymbol{t}_2 \\
\widetilde{\boldsymbol{m}}_1 &= \left(\cos\alpha\sin\psi\cos\theta + \sin\alpha\cos\psi\right)\boldsymbol{m}_2 \\
&\quad + \left(\cos\alpha\cos\psi\cos\theta - \sin\alpha\sin\psi\right)\widetilde{\boldsymbol{m}}_2 \\
&\quad - \cos\alpha\sin\theta\boldsymbol{t}_2 \\
\boldsymbol{t}_1 &= \sin\psi\sin\theta\boldsymbol{m}_2 + \cos\psi\sin\theta\widetilde{\boldsymbol{m}}_2 + \cos\theta\boldsymbol{t}_2
\end{aligned} \tag{s10}$$

Using the relations (s9) we derive analytical expressions (s11) of vectorial products involve in the expression of bending torque $\mathbf{\Gamma}_b$ and twisting torque $\mathbf{\Gamma}_t$ in term of Euler angles and material frame of body 1.

$$\begin{aligned}
\boldsymbol{m}_1 \otimes \boldsymbol{m}_2 &= \left(\cos\psi\sin\alpha + \cos\theta\cos\alpha\sin\psi\right)\boldsymbol{t}_1 - \sin\psi\sin\theta\widetilde{\boldsymbol{m}}_1 \\
\widetilde{\boldsymbol{m}}_1 \otimes \widetilde{\boldsymbol{m}}_2 &= \left(\sin\psi\cos\alpha + \cos\theta\sin\alpha\cos\psi\right)\boldsymbol{t}_1 + \cos\psi\sin\theta\boldsymbol{m}_1 \\
\boldsymbol{t}_1 \otimes \boldsymbol{t}_2 &= \sin\theta\sin\alpha\widetilde{\boldsymbol{m}}_1 + \sin\theta\cos\alpha\boldsymbol{m}_1
\end{aligned} \tag{s11}$$

Using expression (s11) and after some calculus we obtain the expression of twisting torque (s12).

$$
\begin{aligned}
\boldsymbol{\Gamma}_t &= -\left(\boldsymbol{t}_1 \otimes \nabla_{\boldsymbol{t}_1} + \boldsymbol{m}_1 \otimes \nabla_{\boldsymbol{m}_1} + \widetilde{\boldsymbol{m}}_1 \otimes \nabla_{\widetilde{\boldsymbol{m}}_1}\right) E_t \\
&= \frac{g_t|\phi|}{(1+\cos\theta)\,|\sin\phi|}\left(\boldsymbol{m}_1 \otimes \boldsymbol{m}_2 + \widetilde{\boldsymbol{m}}_1 \otimes \widetilde{\boldsymbol{m}}_2 - \cos\phi\boldsymbol{t}_1 \otimes \boldsymbol{t}_2\right) \\
&\propto \left(\cos\psi\sin\alpha + \cos\theta\cos\alpha\sin\psi + \sin\psi\cos\alpha + \cos\theta\sin\alpha\cos\psi\right)\boldsymbol{t}_1 \\
&\quad - \left(\sin\psi\sin\theta + \cos\left(\psi+\alpha\right)\sin\theta\sin\alpha\right)\widetilde{\boldsymbol{m}}_1 \\
&\quad + \left(\cos\psi\sin\theta - \cos\left(\psi+\alpha\right)\sin\theta\cos\alpha\right)\boldsymbol{m}_1 \\
&\propto \sin\left(\alpha+\psi\right)\left(1+\cos\theta\right)\boldsymbol{t}_1 \\
&\quad - \sin\theta\left(\sin\psi + \cos\psi\cos\alpha\sin\alpha - \sin\psi\sin^2\alpha\right)\widetilde{\boldsymbol{m}}_1 \\
&\quad + \sin\theta\left(\cos\psi - \cos\psi\cos^2\alpha + \sin\psi\sin\alpha\cos\alpha\right)\boldsymbol{m}_1 \\
&\propto \sin\left(\alpha+\psi\right)\left(1+\cos\theta\right)\boldsymbol{t}_1 \\
&\quad - \sin\theta\left(\sin\psi\cos^2\alpha + \cos\psi\cos\alpha\sin\alpha\right)\widetilde{\boldsymbol{m}}_1 \\
&\quad + \sin\theta\left(\cos\psi\sin^2\alpha + \sin\psi\sin\alpha\cos\alpha\right)\boldsymbol{m}_1 \\
&\propto \sin\left(\alpha+\psi\right)\left(1+\cos\theta\right)\boldsymbol{t}_1 \\
&\quad - \sin\left(\alpha+\psi\right)\sin\theta\cos\alpha\widetilde{\boldsymbol{m}}_1 \\
&\quad + \sin\left(\alpha+\psi\right)\sin\theta\sin\alpha\boldsymbol{m}_1 \\
&= \frac{g_t|\phi|\sin\phi}{(1+\cos\theta)\,|\sin\phi|}\left(\boldsymbol{t}_1 + \boldsymbol{t}_2\right) \\
\beta\boldsymbol{\Gamma}_t &= \frac{g_t\phi}{1+\cos\theta}\left(\boldsymbol{t}_1 + \boldsymbol{t}_2\right) \quad \text{with} \quad \phi \in \left]-\pi;\pi\right[\quad \text{and} \quad \theta \in \left[0;\pi\right[
\end{aligned} \tag{s12}
$$

Supplementary Movies

Movies are available at the following url:

`http://vimeo.com/51918121`:
Global versus local thermostat

`http://vimeo.com/51918378`:
DNA under traction force of 0.74 pN and fixed linking number $Lk = 15$

`http://vimeo.com/51918151`:
DNA under traction force of 0.74 pN and fixed twisting torque $\Gamma = 15$ pN $\cdot$ nm
